# Incomplete lineage sorting impacts the inference of macroevolutionary regimes from molecular phylogenies when concatenation is employed: An analysis based on Cetacea

**DOI:** 10.1002/ece3.4212

**Published:** 2018-06-11

**Authors:** Anieli G. Pereira, Carlos G. Schrago

**Affiliations:** ^1^ Department of Genetics Federal University of Rio de Janeiro Rio de Janeiro Brazil

**Keywords:** concatenation, diversification, multispecies coalescent, simulation

## Abstract

Interest in methods that estimate speciation and extinction rates from molecular phylogenies has increased over the last decade. The application of such methods requires reliable estimates of tree topology and node ages, which are frequently obtained using standard phylogenetic inference combining concatenated loci and molecular dating. However, this practice disregards population‐level processes that generate gene tree/species tree discordance. We evaluated the impact of employing concatenation and coalescent‐based phylogeny inference in recovering the correct macroevolutionary regime using simulated data based on the well‐established diversification rate shift of delphinids in Cetacea. We found that under scenarios of strong incomplete lineage sorting, macroevolutionary analysis of phylogenies inferred by concatenating loci failed to recover the delphinid diversification shift, while the coalescent‐based tree consistently retrieved the correct rate regime. We suggest that ignoring microevolutionary processes reduces the power of methods that estimate macroevolutionary regimes from molecular data.

## 1 INTRODUCTION

Evolutionary studies are recently abounding with analyses that use molecular phylogenies to investigate macroevolutionary problems related to lineage diversification rates. The number of methods proposed to pursue this task has increased significantly, all relying on time‐calibrated phylogenetic trees with extant species, adopting both likelihood and Bayesian frameworks to estimate the number, magnitude, and locations of diversification rate shifts (Alfaro et al., 2009; Rabosky, 2014; Rabosky & Huang, 2016; Rabosky et al., 2014). In parallel, over the last few years, the inference of molecular phylogenies itself has been transformed at its very core by the development of the multispecies coalescent theory (Edwards, 2009; Xu & Yang, 2016), which explicitly separates the statistical process that models the species tree (speciation) from the process that models gene genealogies in such trees (multispecies coalescent). These two novel theoretical developments, however, are rarely considered jointly (Degnan & Rosenberg, 2009; Song, Liu, Edwards, & Wu, 2012). This is problematic because the estimation of macroevolutionary speciation rates should be carried out using the species tree instead of gene trees, which is the standard practice (Song et al., 2012).

Theoretically, employing gene trees rather than the species tree to estimate macroevolutionary parameters may lead to significant bias; especially in shallow divergences, where incomplete lineage sorting (ILS) has a relatively larger impact on species divergence times (Angelis & Dos Reis, 2015; Leaché, Harris, Rannala, & Yang, 2014). This issue is not ameliorated using the tree built from concatenating genes into a supermatrix because even if the phylogeny is correctly determined, the biological meaning of branch lengths in such trees is elusive, as the age of nodes do not mirror speciation times (Angelis & Dos Reis, 2015; Edwards & Beerli, 2000). Population‐level phenomena, such as ILS, gene duplication and loss, and hybridization will increase the probability of gene tree/species tree mismatch (Degnan & Rosenberg, 2009; Liu, Yu, Pearl, & Edwards, 2009; Schrago, Menezes, Furtado, Bonvicino, & Seuanez, 2014; Song et al., 2012; Tonini, Moore, Stern, Shcheglovitova, & Ortí, 2015).

Even though, the standard practice when studying lineage diversification rates is to rely on molecular phylogenies inferred from concatenated loci (Arbour & Santana, 2017; Shi & Rabosky, 2015). Concatenation ignores differences in evolutionary histories between loci, which are useful to inform on ancestral population dynamics, allowing the decouple of the processes that generate the phylogeny and gene trees (Liu, Yu, Kubatko, Pearl, & Edwards, 2009). Moreover, phylogeny inference from concatenated genomic regions without accounting for conflicting histories is a case of model misspecification, which has been shown to impact estimates of macroevolutionary parameters (Revell, Harmon, Glor, & Linder, 2005). Previous studies demonstrated that concatenating sequences may lead to biased estimates of the species phylogeny (Edwards et al., 2016; Liu, Xi, & Davis, 2015). Accounting for variation in gene genealogies in a population‐level framework while estimating phylogenies is computationally demanding, and heuristic approaches were devised. However, the performance of such approaches has been questioned (Gatesy & Springer, 2013), and further investigation is required. Notwithstanding, it is desirable that biologists should account for population genetics principles, modeled by the multispecies coalescent process, while estimating species trees, and associated macroevolutionary diversification parameters, from biological sequences. To date, the consequences of adopting gene trees rather than the species tree in macroevolutionary inference have not been thoroughly examined.

This prompted us to evaluate the impact of incomplete lineage sorting on the inference of macroevolutionary regimes. To do so, we employed the widely used Cetacea phylogeny of Steeman et al. (2009), which is regarded as an exemplary case of a tree topology containing an evolutionary radiation (family Delphinidae). We investigated the performance of phylogenies obtained by both concatenation and the multispecies coalescence in recovering such a macroevolutionary regime.

## 2 MATERIALS AND METHODS

### 2.1 Simulating gene trees and alignments

To investigate the effects of incomplete lineage sorting on the inference of macroevolutionary parameters, we simulated gene trees, as well as their respective sequence alignments, using the time‐dated phylogeny (used here as the species tree) of cetaceans provided by Steeman et al. (2009), hereafter S09, which encompasses 98% of the extant cetacean diversity. This phylogeny was employed because it has been repeatedly used as a study case in recent analyses, where a shift of macroevolutionary regime for higher diversification rates during the early diversification of dolphins was unambiguously recovered (Rabosky, 2014). Although the S09 time‐dated tree was not originally inferred using coalescent‐based methods; we used it as a template to investigate macroevolutionary estimation because the occurrence of a diversification rate shift in stem Delphinidae has not been questioned so far.

**Figure 1:**
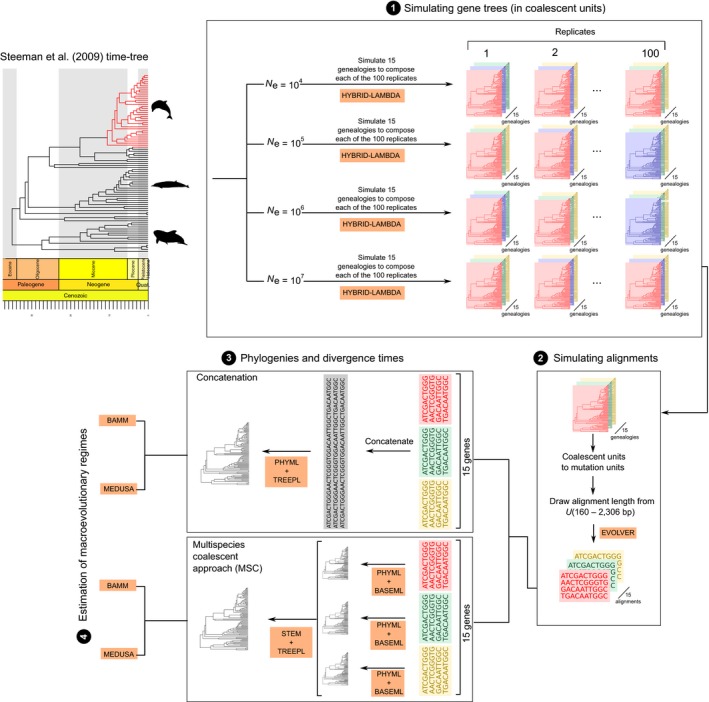
Summary of the simulation steps used to generate data for evaluating the impact of population processes on the estimation of diversification rates and shifts. Steeman et al. (2009) time‐tree, which contains a diversification shift near the diversification of delphinids, was used as a template to generate gene genealogies under four different population size scenarios. A total of 100 replicates, with 15 gene genealogies each, was produced (1). Alignments were simulated after transforming branch lengths of gene trees from coalescent units to mutation units and by sampling an alignment length from an empirically‐driven uniform distribution (2). Phylogenies and divergence times were then estimated using simulated alignments by employing both concatenation and a MSC approach (3). The inferred dated phylogenies were finally used to estimate the shift of macroevolutionary regime (4)

Gene trees were simulated under the multispecies coalescent model using four increasing ancestral effective population sizes: *N*_e_ = 10^4^, 10^5^, 10^6^, and 10^7^ Wright‐Fisher individuals, which resulted in four independent datasets. For each population size dataset, we composed 100 replicates containing 15 bifurcating gene trees that were evolved employing HYBRID‐LAMBDA software (Zhu, Degnan, Goldstein, & Eldon, 2015). Branch lengths in simulated gene trees, which were originally in coalescent units (2*N*_e_ generations), were transformed into mutation units using the genomic rate of 10^‐8^ mutations/site/generation and assuming generation time = 10 years, which is close to the mean generation time of delphinids, 9.6 years (Pacifici et al., 2013). Mutation rate per generation was based on the human estimates obtained from genome‐wide studies (Kong et al., 2012; Lipson et al., 2015). Gene trees in mutation units were then used to simulate gene alignments with the EVOLVER program of the PAML package (Yang, 2007) under the Jukes‐Cantor substitution model. Alignment lengths of each gene (locus) were sampled from a uniform distribution from 160 to 2306 bp, which corresponds to the minimum and maximum lengths of genes used in Steeman et al. (2009). Therefore, for each *N*_e_ value, we obtained 1,500 independent alignments. A summary of the entire simulation procedure is found in Figure 1.

### 2.2 Phylogenies and divergence times

To mimic the procedure carried out by researchers when inferring macroevolutionary regimes, for each population size dataset, phylogenies and divergence times were estimated from simulated alignments. When inferring phylogenies, we used two competing approaches commonly implemented in recent literature: concatenation and species tree inference based the multispecies coalescent (MSC) theory. For both approaches, maximum likelihood trees were estimated in PhyML 3 (Guindon et al., 2010) under the Jukes‐Cantor model. Because the coalescent program STEM requires ultrametric trees, and as sequence data were simulated under a single substitution rate, branch lengths in PhyML trees were re‐estimated with the program BASEML of the package PAML 4, enforcing the molecular clock (Yang, 1997, 2007). Divergence time estimation was conducted in treePL (Smith & O’Meara, 2012). The algorithm was run without cross‐validation and setting the smooth parameter = 0.1. Five calibrations were used according to the fossils and ages employed in S09, plus a maximum limit of 40 Mya to the age of crown Cetacea based on Chen, Xu, Zhou, and Yang (2011) (Table 1). Species tree estimation under the MSC model was implemented in STEM (Kubatko, Carstens, & Knowles, 2009) by fixing θ values from 0.0004 (*N*_e_ = 10^4^) to 0.4 (*N*_e_ = 10^7^), respectively. As means of evaluating the accuracy and precision of inferred trees, for each replicate, we calculated the topological distance from the estimated trees to the S09 phylogeny using the Penny and Hendy (1985) metric implemented in the R package ‘ape’ (Paradis, Claude, & Strimmer, 2004) (Figure 1).

**Table 1:** Fossil calibrations used in divergence time estimation

Divergence	Age (Ma)
*B. mysticeti* and *D. delphis*	33.3–40 Ma
*B. physalus* and *M. novaeanglia*	>7.3 Ma
*L. vexillifer* and *D. delphis*	>23.5 Ma
*I. geoffrensis* and *P. blainvillei*	>12 Ma
*D. leucas* and *P. phocoena*	>10 Ma

### 2.3 Estimation of macroevolutionary regimes

The dynamics of species diversification was inferred for all dated phylogenies obtained from concatenation and the MSC method using the widely employed software in the literature, namely BAMM (Rabosky, 2014) and MEDUSA (Alfaro et al., 2009; Pennell et al., 2014). BAMM accounts for the variation in diversification rates through time and among lineages using transdimensional (reversible‐jump) Markov chain Monte Carlo (rjMCMC) (Rabosky, 2014). In BAMM, Markov chains were sampled every 1,000th generation until 7,500 trees were collected after a burn‐in of 25%. Prior distributions were set according to the *setBAMMPriors* function from the BAMMtools R package (Rabosky et al., 2014). MEDUSA assumes constant diversification rates through time to detect rate variations in lineages based on a maximum likelihood approach. We used the birth‐death (BD) model to run MEDUSA analyses.

When evaluating the performance of trees estimated using either concatenation or the MSC‐based method in recovering the same macroevolutionary regime inferred for the S09 tree (Steeman et al., 2009), we measured (a) the number of shifts inferred on the tree (BAMM and MEDUSA); (b) the marginal posterior probability for 0, 1 and 2‐diversification rate shifts regimes in BAMM; and (c) the age of the inferred diversification shift (BAMM and MEDUSA). Item (iii) was evaluated because, in addition to correctly inferring the number of diversification shifts, we expect the age in which the change of macroevolutionary regime took place to be correctly recovered.

## 3 RESULTS AND DISCUSSION

**Figure 2:**
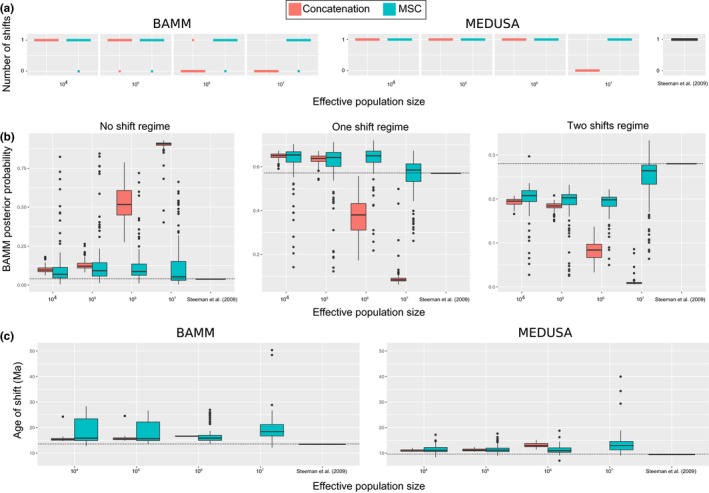
Impact of the concatenation and MSC methods in the inference of macroevolutionary rates. (a) The number of shifts inferred; (b) the posterior probability for 0‐, 1‐ and 2‐shift configurations in simulated datasets estimated in BAMM; and (c) ages of the inferred diversification rate shifts. In all panels, results obtained using the original Steeman et al. (2009) tree is also displayed for the sake of comparison

We found that under smaller effective population sizes (10^4^ and 10^5^), most macroevolutionary analyses of BAMM and MEDUSA correctly recovered one diversification rate shift, independent of the tree estimation method. As population sizes increased, however, only analyses conducted using the MSC‐based trees recovered a rate shift in BAMM and MEDUSA. For instance, when *N*_e_ = 10^7^, phylogenies estimated by concatenation were unable to inform on the presence of a shift of macroevolutionary regime (0%) (Figure 2a). In BAMM, we draw the marginal posterior densities for macroevolutionary regimes with a varying number of shifts. The posterior probability of no diversification shift was low under smaller *N*_e_ (10^4^ and 10^5^), which correctly captured the empirical scenario of a 1‐shift (Figure 2b). Under large *N*_e_, however, only datasets in which the MSC‐based trees were used rendered a low probability of a 0‐shift. Trees from concatenated alignments consistently yielded >70% probability for the incorrect 0‐shift configuration. Posterior densities for the regime with one diversification rate shift indicated that concatenation failed to recover the cetacean macroevolutionary regime under *N*_e_ = 10^6^ and 10^7^, whereas posteriors for the 1‐shift configuration from the MSC‐based trees rendered a higher probability even under large effective population sizes (Figure 2b).

Under *N*_e_ = 10^4^ and 10^5^, the age of the Cetacea diversification rate shift was correctly inferred independent of the tree building approach (Figure 2c). When MSC trees were employed, however, a greater variance of the age was retrieved. Theory predicts that the mean coalescent time, that is, genetic divergence between two lineages, will increase with increasing population size because the number of substitutions accumulated in the ancestral species equals the scaled population parameter θ = 4*N*_e_μ. MSC methods thus differentiate between the mean coalescent time (genetic divergence) and the speciation time *per se* (reproductive isolation) of lineages. Therefore, as expected, when *N*_e_ = 10^6^, the ages the delphinid macroevolutionary regime shift inferred from trees estimated using concatenation were older when compared to MSC‐based trees. When *N*_e_ = 10^7^, the comparison could not be performed, as only analyses using the MSC‐based trees recovered a shift. Differences between the ages of shifts inferred by BAMM and MEDUSA might be explained by the fact that MEDUSA assigns the location of diversification rate shifts to nodes rather than along branches (Alfaro et al., 2009; Rabosky, 2014).

**Figure 3:**
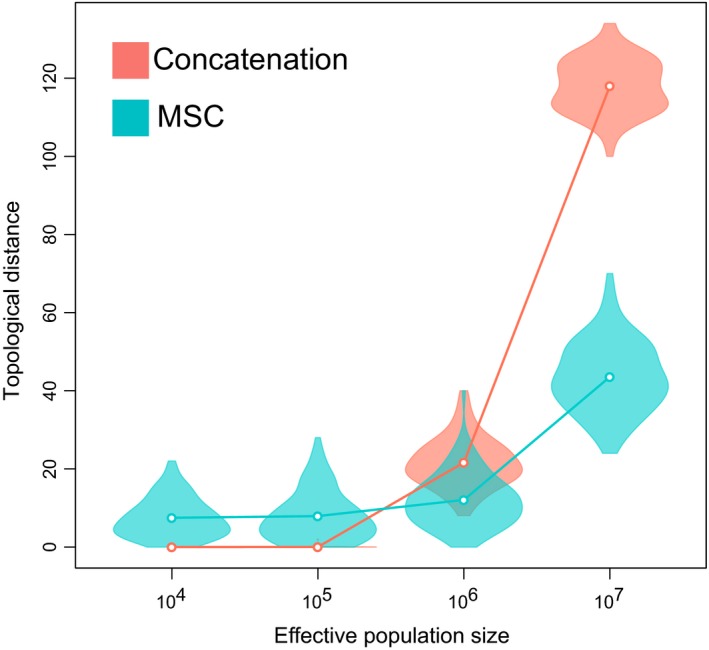
Association between topological distances of the inferred tree using concatenation or MSC and the effective population size. Violin plots depict the distribution of the topological distances of the phylogenies estimated from each tree‐building method across the 100 replicates. Circles indicate the mean topological distance for replicates in each population size scenario

Ultimately, the inference of macroevolutionary regimes from molecular phylogenies evidently depends on the correct estimation of both topology and time‐transformed branch lengths. In our study, as the effective population size increased, topological distances between the phylogenies inferred assuming the MSC trees and S09 tree were smaller than those calculated using concatenation (Figure 3). This corroborates the findings that the MSC‐based methods are more accurate in recovering the true phylogeny under strong ILS caused by large effective population sizes (Edwards, Liu, & Pearl, 2007; Kubatko & Degnan, 2007; Kubatko et al., 2009).

Studies have highlighted the difficulty of estimating macroevolutionary parameters from molecular phylogenies (Quental & Marshall, 2010). Moreover, recent works suggested that the methodological approaches implemented in both BAMM and MEDUSA may be flawed (May & Moore, 2016; Moore, Höhna, May, Rannala, & Huelsenbeck, 2016). Although methodological improvements were already implemented (Rabosky, Mitchell, & Chang, 2017), it is conceivable that dated molecular phylogenies alone are insufficient to estimate the shifts of macroevolutionary regimes reliably. In this sense, our study is an additional alert that the methodological framework used to infer the molecular phylogeny itself should account for population‐level processes, such as incomplete lineage sorting. Future investigation of the impact of molecular phylogenies on estimating lineage diversification rates should be ideally conducted under the multispecies coalescent model.

Using the cetacean diversification as a template, our simulations showed that when the effective population size reached 10^6^, phylogenies inferred from concatenation started to differ from the template cetacean tree, whereas coalescent‐based approaches performed better in recovering the template topology. Thus, it is not surprising that under this *N*_e_ value, the inference of the diversification rate regime from coalescent‐based trees outperformed concatenation in most simulations. Evidently, we should evaluate the biological relevance of such findings by contrasting our results with empirically estimated effective population sizes. Oliver (2013) compiled a list of effective population size estimates to show that microevolutionary processes may affect deep‐time phylogenetic relationships. Effective population sizes as large as 10^6^ have been frequently recovered for invertebrates (Papadopoulos, Peijnenburg, & Luttikhuizen, 2005), plants (McDaniel et al., 2010), and even vertebrates (Carneiro, Ferrand, & Nachman, 2009). Although rarer, *N*_e_ values greater than 10^7^ were indeed reported for mollusks (7.2 × 10^7^) (Etter et al., 2011), insects (~2.0 × 10^7^) (Schoville, Stuckey, & Roderick, 2011) and birds (1.6 × 10^7^_)_ (Maley & Winker, 2010). Our results demonstrated that diversification rate inference in such lineages must rely on coalescent‐based tree inference. In such cases, the use of trees inferred from concatenated genes will increase Type II error because the probability of identifying a diversification rate shift decreases significantly. In the case of cetaceans, *N*_e_’s varied from values near 10^4^ (e.g., 1,218 for bottlenose dolphins, (Viaud‐Martinez, Brownell, Komnenou, & Bohonak, 2008) to 214,629 for harbor porpoises (Viaud‐Martínez et al., 2007). Therefore, the range of *N*_e_ values used in our simulations included the values measured for cetaceans. Our simulations showed that, within this range, phylogenies inferred from both concatenation and coalescent‐based approaches retain the information required to correctly estimate macroevolutionary regime shifts.

We suggest that further developments in macroevolutionary rate estimation should not overlook the effects of microevolutionary processes on phylogenies. Although our simulations tried to capture the intricacies of empirical data manipulation, they were still based on simplistic assumptions, such as a constant effective population size along the phylogeny. Moreover, it would be meaningful to quantify the effects of tree topology shapes that comprise differing mixtures of deep and shallow divergences. If MSC is disregarded, topologies containing both intra‐ and inter‐species diversity are difficult to model, as they contain both the coalescent and speciation processes.

## AUTHORS CONTRIBUTION

AGP and CGS conceived and designed the study, performed all analyses, interpreted the results and wrote the manuscript.

## DATA ACCESSIBILITY STATEMENT

All phylogenies used in this study were previously published and original sources were referenced in the text. Data generated by simulation are archived in the Dryad repository: https://doi.org/10.5061/dryad.np7332q
